# Effects of Food Changes on Intestinal Bacterial Diversity of Wintering Hooded Cranes (*Grus monacha*)

**DOI:** 10.3390/ani11020433

**Published:** 2021-02-07

**Authors:** Nazhong Zhang, Lizhi Zhou, Zhuqing Yang, Jingjing Gu

**Affiliations:** 1 School of Resources and Environmental Engineering, Anhui University, Hefei 230601, China; x18201012@stu.ahu.edu.cn (N.Z.); x18201023@stu.ahu.edu.cn (Z.Y.); x18301087@stu.ahu.edu.cn (J.G.); 2 Anhui Province Key Laboratory of Wetland Ecosystem Protection and Restoration, Anhui University, Hefei 230601, China

**Keywords:** food composition, intestinal bacteria, high-throughput sequencing, hooded crane

## Abstract

**Simple Summary:**

The intestinal microbiota plays a vital role in the health of animals, and food is an important factor that influences the intestinal microbial community. During the winter months, waterbirds require certain foods to supply them with energy through the cold winter. Due to changes in the plant resources available to waterbirds, their intestinal bacteria will vary accordingly. In this study, we analysed the relationship between food composition and intestinal bacteria in hooded cranes (*Grus monacha*). We found that food resources from similar habitats were more similar, and the corresponding hooded crane intestinal bacteria were also more similar. The results show that the intestinal bacteria of hooded cranes had a certain adaptability to the type of food being consumed. This study contributes novel insights into the diet of hooded cranes in the winter months, allowing for improved protection and management strategies.

**Abstract:**

As food is recognised as an important factor affecting the intestinal microbiota, seasonal changes in diet can influence the community composition. The hooded crane (*Grus monacha*) is an endangered migratory waterbird species, with some of the population wintering in the sallow lakes in the middle and lower Yangtze River floodplain. Their food resources have changed seasonally, with a reduction resulting from wetland degradation. To cope with seasonal changes in food availability, hooded cranes must constantly adjust their foraging strategies to survive. We studied the effect of changes in diet on the intestinal bacterial diversity of hooded cranes at Shengjin Lake, using faecal microanalysis and high-throughput sequencing. The results show that the main foods of hooded cranes were *Polygonum criopolitanum, Oryza sativa*, and *Carex* spp., which were significantly related to the composition of the intestinal bacterial community. In addition, foods available from the similar habitats were more similar, and the corresponding hooded crane intestinal bacteria were also more similar. The relative abundance of *Lactobacillus acidipiscis* in January and March was significantly higher than in November. Our research shows that the intestinal bacteria of hooded cranes actively adapt to diet changes to overcome the negative impact of the reduction in food resources, which is vital to the survival of hooded cranes.

## 1. Introduction

Intestinal microorganisms play an important role in maintaining host health by promoting digestion and absorption, fat metabolism, immune regulation, vitamin synthesis, and other host functions [1]. The intestinal microbial community is in a state of balance between animals and their surrounding environment. However, resource characteristics under different environmental conditions, especially food resources, will affect the host microbiota composition, and will cause animals to adjust their intestinal microbial flora to adapt to changes in food availability.

Diet is one of the most relevant factors affecting intestinal microbial communities. Food not only provides energy and structural components, but also contains carbon and nitrogen sources, which are essential elements for the growth and metabolism of intestinal microbes [2]. Changes in dietary patterns can directly affect the composition and function of the intestinal microbial community through changes in food quantity and micronutrients in the gut. For example, in order to digest the large amount of cellulose in bamboo, pandas harbour large numbers of Firmicutes in their intestines [3]. Significant differences were found among the intestinal flora of carnivores, omnivores, and vegetarians, and the microbial diversity increased in sequence [4]. The intestinal microbiome can respond quickly to changes in diet. The short-term intake of animal or plant diets will not merely affect the structure, function, and metabolic activities of the gut microflora, but also microorganisms in the diet will temporarily settle in the gut, which can even temporarily overwhelm individual differences in microbial gene expression [5]. The hindgut of vultures with less microbial diversity is dominated by Clostridia and Fusobacteria, which are common soil bacteria and potential pathogens to most vertebrates, and which may be contributed by food sources [6].

The hooded crane (*Grus monacha*) is an important representative of wetland wildlife, is a key protected species in China, and is listed as a vulnerable (VU) species on the International Union for Conservation of Nature and Natural Resources (IUCN) Red List of Threatened Species [7]. Shengjin Lake is an internationally recognised wetland in Anhui Province, China, located in the middle and lower Yangtze River floodplain. It is the key wintering ground for hooded cranes in China, with approximately 300 hooded cranes flocking to the area in winter [8]. In China, previous studies found that hooded cranes mainly feed on *Zea mays* and animal food in Lindian [9]. At Poyang Lake, they mainly feed on *Polygonum criopolitanum*, *Potentilla imprichtii*, *Carex tristachya*, and *Tulipa edulis* [10], whereas, at Shengjin Lake, the early wintering hooded cranes mainly consume *Vallisneria natans* and *Potamogeton malaianus* [11]. Later in the season, hooded cranes mainly feed on *Oryza sativa*, *Potentilla supina*, and *P. criopolitanum* [12]. Currently, due to the degradation of wetlands, submerged plants at Shengjin Lake, such as *V. natans*, have been greatly reduced. During the winter months, the reduced food supply forces hooded cranes to change their foraging patterns [13].

In winter, hooded cranes are omnivorous, but mainly feed on plants. As dietary changes affect the intestinal bacterial community, it was assumed that the composition of the intestinal bacterial community of hooded cranes at Shengjin Lake would change with the variation in diet as winter progressed. However, there are few studies on the intestinal microbes of hooded cranes, specifically related to food composition and the intestinal bacterial community. In the present study, the plant foods and gut microbiomes of the wintering cranes at Shengjin Lake were evaluated by microscopy and 16S ribosomal RNA (rRNA) high-throughput sequencing technology. So as to understand whether the intestinal bacteria of hooded cranes respond positively to changes in the available food, we (a) studied the composition of foods available and intestinal bacterial community, (b) evaluated the correlation between foods available and intestinal bacterial community composition, and (c) compared the alpha and beta diversity of foods and intestinal bacteria.

## 2. Materials and Methods

### 2.1. Ethics Statement

We obtained permission from the Anhui Shengjin Lake National Nature Reserve and collected faecal samples of hooded cranes after foraging to avoid human disturbance. Non-invasive sampling did not involve hunting experimental animals [14].

### 2.2. Study Site and Sample Collection

The research site at Shengjin Lake (30°15′–30°28′ north (N), 116°58′–117°14′ east (E)) in Anhui Province, China, is rich in natural resources. As Shengjin Lake is a key wintering habitat for hooded cranes in China, it was selected as the research site (File S1, Figure S1, Supplementary Materials).

We used non-invasive sampling methods to collect the fresh faeces of wintering hooded cranes at Shengjin Lake on 23 November 2018, 9 January 2019, and 8 March 2019. A total of 54 faecal samples were used to analyse the food composition and intestinal bacteria of the hooded cranes. Among them, 19 samples were collected from the grass land (30°20′34.41″ N, 117°0′24.31″ E) in November, 20 samples were collected from the rice field (30°21′10.85″ N, 116°59′6.76″ E) in January, and 15 samples were collected from the grass land (30°20′9.4″ N, 117°0′58.43″ E) in March.

Before collecting samples, we used binoculars and monoculars to observe the concentrated foraging area of hooded cranes. A larger group was chosen to ensure that there were no other cranes, geese, or ducks within 50 m during the foraging period. Hooded cranes left the foraging site after consuming food, and we immediately went to the foraging site to collect faecal samples. The foraging locations were confirmed by the footprints and foraging pits left by the hooded cranes. To avoid sample contamination, one pair of plastic gloves was used for each sample, and the interval distance between samples was more than 5 m to avoid pseudoreplication [15]. All faecal samples were immediately stored in an incubator with ice bags for short-distance transportation, stored immediately at −80 °C after delivery to the laboratory. When conducting formal experiments, the inner core of the faeces was used to prevent contamination from the outside.

### 2.3. Sample Pretreatment

We describe the four sections of sample DNA extraction, species identification, PCR, and amplicon library preparation in File S2 (Supplementary Materials).

### 2.4. Sequence Data Processing

QIIME (v.2-2020.2) [16] was used to process raw data. We filtered the poor-quality sequences using the deblur algorithm. Sequences were grouped into amplicon sequence variants (ASVs) [17]. The VSEARCH method was used to filter chimeras, and the UNITE database (20 February 2020) was applied for annotating Taxonomy to each ASV [18]. Singletons were filtered for downstream analysis. We compared bacterial community for all samples using a subset of 5226 sequences per sample.

### 2.5. Determination of Potentially Pathogenic Species

All identified bacterial species were retrieved from the Web of Science database to search for potentially pathogenetic species (File S3, Supplementary Materials). Further research has been carried out on potentially pathogenic in humans or other animals.

### 2.6. Testing Food Composition

In this experiment, plant proportions in the faecal samples were analysed by means of faecal microanalysis. The concentrated nitric acid method [12] was used to make the microslides. The steps were as follows:

1. Drying: The faecal samples were placed in an oven at 65 °C until the weight was constant.
2. Grinding: Dried faecal samples were ground into powder and placed in self-sealing bags.
3. Screening: The ground faecal samples were screened with a net screen of 40 mesh (0.40 mm) and 100 mesh (0.15 mm) to make the size of sample fragments between 0.15 mm and 0.40 mm.
4. Concentrated nitric acid treatment: Sieving substances on a 100 mesh net screen were placed into a 50 mL beaker. Then, 2–3 mL of concentrated nitric acid was added to the small beaker. The mixture was kept quiescent for 3 min before being heated in a water bath at 90 °C for 2–3 min. The mixture was diluted with water, poured through a sieve, rinsed with distilled water until the colour was constant, and placed into a petri dish.
5. Slide generation: A small amount of sample fragment was placed on a glass slide with a glue head dropper. A drop of distilled water was placed on the sample, which was unfolded with tweezers, before it was covered with a coverslip; excess water was removed.

One hundred fields of view were observed for each faecal sample under 10 magnification, and species identification was conducted by referring to the established plant cell morphological atlas database (The experiment method was the same as above). The names of recognisable plant fragments in each field of vision were noted, and plants that appeared in one field of vision are counted as one. The recorded data were input into an Excel 2007 worksheet, to obtain the frequency (F) of each plant (F represents the frequency of each plant in 100 fields of view), average density (Di), and relative density (RD). F and RD were used as the criteria for crane food composition [10,12].

Average density: Di = −ln (1 − F/100).(1)

Relative density: RD = (Di/∑Di) × 100%.(2)

According to the RD value, the food was divided into three categories: RD ≥ 10% represents the main food type, 1% ≤ RD < 10% represents a common food type, and RD < 1% represents an occasional food type.

### 2.7. Data Analysis

We used the Mantel test to evaluate the correlation between food composition and intestinal bacteria. A Kolmogorov–Smirnov test was applied to analyse whether the data followed a normal distribution (*p* > 0.05, normal; *p* ≤ 0.05, non-normal). Alpha diversity analysis (ASV richness, Chao1, Simpson, Shannon) was evaluated using a one-way ANOVA. The one-way ANOVA was used to deal with normally distributed data. If the differences were significant (*p* < 0.05), we used the Tukey honestly significant difference (HSD) test (*p* < 0.05) to perform post hoc pairwise multiple comparisons. The nonparametric Kruskal–Wallis test (*p* < 0.05) was used for non-normally distributed data and post hoc pairwise multiple comparisons were performed by Dunn–Bonferroni test. These methods were applied for analysing the relative abundance of dominant bacteria (>1%) and alpha diversity across all samples. Generally, *p* < 0.05 was deemed significant and *p* < 0.01 represented a high level of significance (File S1, Table S1, Supplementary Materials). Nonmetric multidimensional scaling (NMDS) was applied to evaluate beta diversity. The analysis of similarity (ANOSIM, permutations = 999) [19] was performed using the vegan package in R (V.3.4.4) to compare the groups in each month. Similarity percentage (SIMPER) analysis was used to compare the differences in ASVs across each sampling month [20]. The Labdsv package was applied to analyse the indicator bacterial species. A nonparametric Kruskal–Wallis rank-sum test (alpha value: 0.05; effect size threshold: 2) for biomarker identification was used to evaluate linear discriminant analysis (LDA) effect sizes (LEfSe) to rank the most abundant modules in each month [21]. The Galaxy workflow was used to perform LEfSe, and diversities were expressed as the mean ± SD. The raw data of intestinal bacteria in hooded cranes were submitted to the National Centre for Biotechnology Information (NCBI) Sequence Read Archive (BioProject identifier (ID): PRJNA687921).

## 3. Results

### 3.1. Food Composition

**Figure 1:**
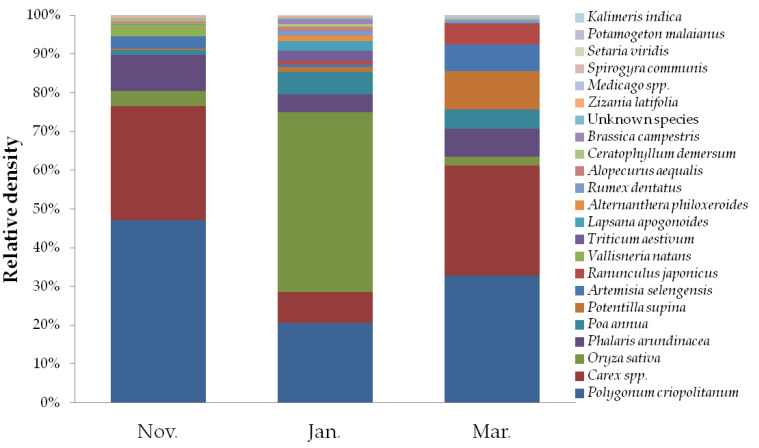
Relative density of foods of hooded cranes in the 3 months.

Through a comparison with the plant cell morphological atlas database, the faecal samples of hooded cranes from Shengjin Lake revealed a total of 20 species and 22 genera from 13 families, among which plants of *Carex* and *Medicago* were only identified to the genus level. In these plants, common genera were *Polygonum*, *Carex*, and *Oryza*, and common families were Polygonaceae, Cyperaceae, and Poaceae. According to the RD values, there were three main foods, namely, *P. criopolitanum* (33.33% ± 15.09%), *Carex* spp. (21.16% ± 13.75%), and *O. sativa* (19.27% ± 21.61%), which accounted for 73.76% of the total food. *Potenaris arundinacea*, *Poa annua*, *P. supina*, *Artemisia selengensis*, *Ranunculus japonicus*, and *V. natans* represented commonly consumed food, which accounted for 20.49%. The occasional foods included *Triticum aestivum*, *Lapsana apogonoides*, *Alternanthera philoxeroides*, *Rumex dentatus*, *Alopecurus aequalis*, *Ceratophyllum demersum*, *Brassica campestris*, *Zizania latifolia*, *Medicago* spp., *Spirogyra communis*, *Setaria viridi*, *P. malaianus*, *Kalimeris indica*, and some unknown species, accounting for 5.77% of the total food intake. In November, *P. criopolitanum* (47.13% ± 9.01%) and *Carex* spp. (29.36% ± 9.74%) were the main source of food for hooded cranes. In January, *O. sativa* (46.49% ± 7.00%) and *P. criopolitanum* (20.66% ± 5.54%) were the main species consumed. In March, *P. criopolitanum* (32.76% ± 15.09%) and *Carex* spp. (28.48% ± 11.94%) were the main species consumed by hooded cranes (Figure 1 and File S1, Table S2, Supplementary Materials).

### 3.2. Intestinal Bacterial Composition

High-throughput sequencing was performed on the V4–V5 regions of the 16S rRNA of 54 samples during the winter period, and a total of 703,468 sequences were obtained. During the classification process, 1882 representative ASV sequences were obtained. A total of 19 phyla, 48 classes, 88 orders, 145 families, and 235 genera were identified (File S1, Table S3 and Figure S2, Supplementary Materials).

**Figure 2:**
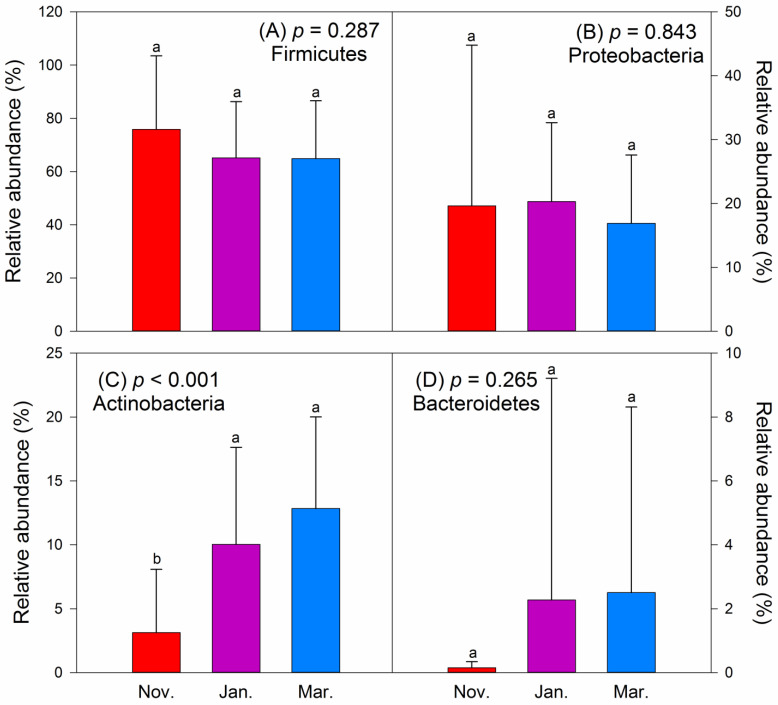
Relative abundance (%) of the dominant intestinal bacterial phyla of hooded cranes in the 3 months. (**A**) Firmicutes; (**B**) Proteobacteria; (**C**) Actinobacteria; (**D**) Bacteroidetes. Letters over the bars indicate pairwise differences according to Tukey honestly significant difference (HSD) test in (**A**–**C**) and Dunn–Bonferroni test in (**D**) at the *p* < 0.05 level. Coloured bars indicate the mean value, and error bars represent the standard deviation.

Four dominant phyla were identified in the intestinal bacterial community of hooded cranes, including Firmicutes (68.84%), Proteobacteria (19.11%), Actinobacteria (8.38%), and Bacteroidetes (1.59%) (File S1, Table S4, Supplementary Materials). The relative abundance of Actinobacteria was significantly lower in November than in January and March, while the other three phyla showed no significant difference across the 3 months (Figure 2). The dominant intestinal bacterial genera were *Lactobacillus* (Lactobacillaceae) (27.08%), *Clostridium* (Peptostreptococcaceae) (9.08%), *Paenibacillus* (Paenibacillaceae) (5.49%), *Clostridium* (Clostridiaceae) (5.27%), *Bacillus* (Bacillaceae) (5.04%), *Methylobacterium* (Methylobacteriaceae) (2.88%), *Martelella* (Aurantimonadaceae) (2.00%), *Enterococcus* (Enterococcaceae) (1.92%), *Escherichia* (Enterobacteriaceae) (1.90%), *Arthrobacter* (Micrococcaceae) (1.80%), *Nocardioides* (Nocardioidaceae) (1.47%), and *Epulopiscium* (Lachnospiraceae) (1.25%) across all samples (File S1, Table S5, Supplementary Materials). The main pathogenic microorganisms were *Escherichia coli* (1.90%), *Clostridium botulinum* (1.14%), and *Enterococcus casseliflavus* (1.00%) (File S1, Table S6, Supplementary Materials). The ASV richness of intestinal pathogenic bacteria was not significantly different (*p* = 0.224) across the three months (File S1, Figure S3, Supplementary Materials).

**Figure 3:**
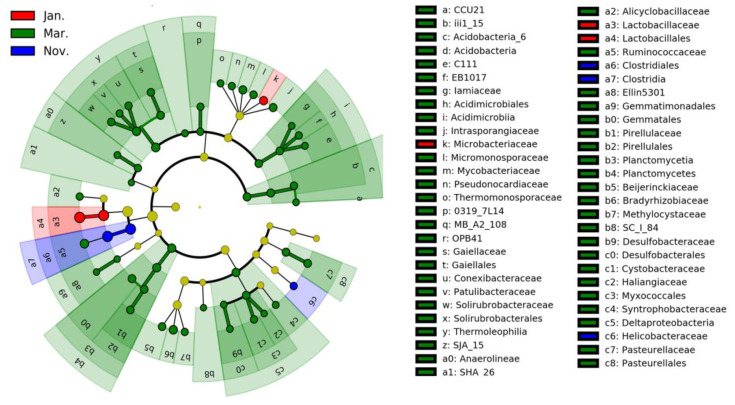
Linear discriminant analysis (LDA) effect size (LEfSe) analysis of the intestinal bacterial biomarkers of hooded cranes among the 3 months. Identified phylotype biomarkers were sorted by effect size and the alpha value was <0.05. Each filled circle represents one biomarker. The cladogram represents the taxonomic hierarchical structure of the phylotype biomarkers identified over the 3 months; blue, phylotypes statistically overrepresented in November; red, phylotypes statistically overrepresented in January; green, phylotypes statistically overrepresented in March.

LEfSe was used to identified specific intestinal bacterial taxa of hooded cranes, and differences were found among them during the three winter months. The results show that the amount of specific bacterial taxa in March was significantly higher than in November and January (Figure 3 and File S1, Figure S4, Supplementary Materials). Variations in the relative abundance of ASV0360 *Lactobacillus acidipiscis* (21.65%) and ASV0001 *Clostridium metallolevans* (11.23%) were primarily responsible for the difference in bacterial community composition in November and January. There were ASV0002 *L. acidipiscis* (12.76%) and ASV0001 *Clostridium metallolevans* (11.92%) between November and March, and ASV0360 *L. acidipiscis* (22.93%) between January and March (Table 1). Indicator analysis identified 21 ASVs, 12, 3, and 6 of which were from November, January, and March, respectively. ASV0001 *C. metallolevans* and ASV0003 Bacillales were the two most abundant indicator species in November, with relative abundances of 8.68% and 7.34%, respectively. The most abundant indicator species of intestinal bacteria in January was ASV0360 *L. acidipiscis*, whose relative abundance was 16.82%. ASV0002 *L. acidipiscis* was the most abundant indicator species in March, with a relative abundance of 7.43% (File S1, Table S7, Supplementary Materials).

### 3.3. Alpha Diversity and Beta Diversity

Mantel test analysis showed that the main foods of hooded cranes have a extremely significant correlation with intestinal bacteria. Among the common foods, *P. arundinacea*, *P. annua, R. japonicus, P. supina*, and *V. natans* had an extremely significant correlation with intestinal bacteria, and *P. criopolitanum and V. natans* had a significant correlation with intestinal potential pathogenic bacteria (Table 2 and Table 3).

**Figure 4:**
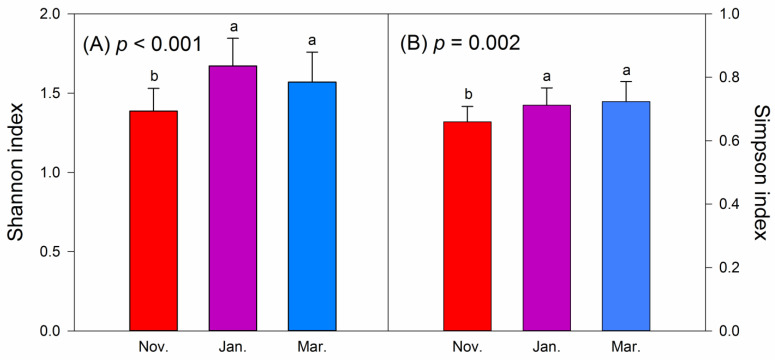
Alpha diversity of foods of wintering hooded cranes in the 3 months. (**A**) Shannon–Weiner index; (**B**) Simpson index. Letters over the bars indicate pairwise differences according to Tukey HSD test at the *p* < 0.05 level. Coloured bars indicate the mean value, and error bars represent the standard deviation.

**Figure 5:**
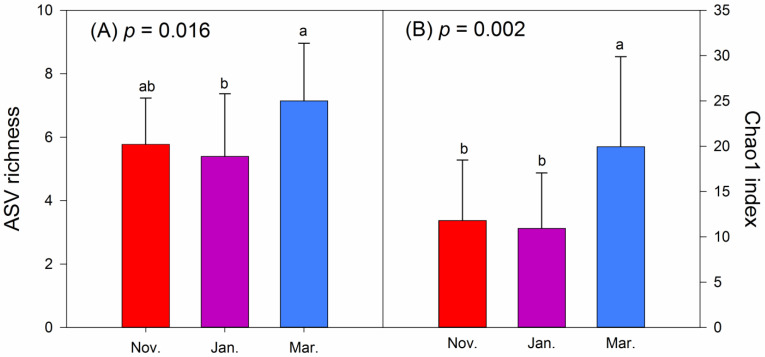
Alpha diversity of intestinal bacteria in wintering hooded cranes in the 3 months. (**A**) ASV richness; (**B**) Chao1 index. Letters over the bars indicate pairwise differences according to Tukey HSD test at the *p* < 0.05 level. Coloured bars indicate the mean value, and error bars represent the standard deviation. ASV, amplicon sequence variants.

The Shannon–Weiner and Simpson indices were applied for evaluating the alpha diversity of foods consumed by hooded cranes (Figure 4). The ASV richness and Chao1 index were applied to assess the alpha diversity of intestinal bacteria (Figure 5). The Shannon (*p* < 0.001) and Simpson (*p* = 0.002) indices indicated significant differences in foods between November and January and between November and March, whereas no significant difference was noted between January and March. The ASV richness (*p* = 0.016) of intestinal bacteria revealed a significant difference between January and March. Moreover, Chao1 (*p* = 0.002) of intestinal bacteria revealed significant differences between November and March and between January and March. Moreover, as winter progressed, the alpha diversity of intestinal bacteria initially decreased before increasing again.

**Figure 6:**
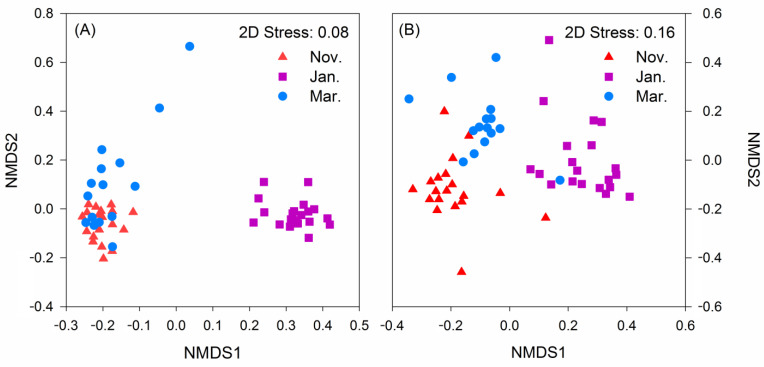
Food composition and intestinal bacterial community compositions of hooded cranes in the 3 months according to nonmetric multidimensional scaling (NMDS). (**A**) Food composition; (**B**) intestinal bacterial community compositions.

There were significant differences in the beta diversity of food composition and the intestinal bacteria of hooded cranes across the 3 winter months. The results of NMDS showed that the food composition and intestinal bacteria of hooded cranes in the same month had a tendency to aggregate (Figure 6). From the similarity analysis test (ANOSIM, analysis of similarities), the food composition and intestinal bacteria of hooded cranes in different months were compared in pairs, and they showed significant differences (Table 4 and Table 5).

## 4. Discussion

Due to an increase in human invasion and interference, wetlands are disappearing at an unprecedented rate, leading to the loss of waterbird habitats. All these factors lead to hooded cranes being under great pressure to survive [22]. In order to relieve the stress of survival, hooded cranes must constantly adjust their foraging strategies [23,24]. Changes in bird foods are an important factor influencing changes in the gut microbial community [25]. In previous studies, the wintering hooded cranes at Shengjin Lake were observed to mainly feed on rice, *O. sativa*, *P. criopolitanum*, and *P. supina* [12]. In this study, we found that the main foods of wintering hooded cranes in November were *P. criopolitanum* and *Carex* spp., in January were *O. sativa* and *P. criopolitanum*, and in March were *P. criopolitanum, Carex* spp., and *O. sativa*. According to the optimal foraging theory, animals preferentially choose foods with higher energy. The wetlands have degraded, and submerged plants have reduced; consequently, the *V. natans* that hooded cranes favour have diminished significantly [26]. A reduction in food supply forces animals to change their foraging patterns [13], which may explain why hooded cranes no longer use *V. natans* as their main food source. During the winter, as food resources vary, cranes have to change their foraging methods [13,23]. In November and March, grasslands are the most abundant and easily available foraging grounds for plants, while, in January, *O. sativa* is the most easily available food resource. Moreover, *P. criopolitanum, Carex* spp., and *O. sativa* all contain higher crude protein and fat [27,28], which may explain why hooded cranes frequently consume these plants.

The dominant phyla of hooded cranes intestinal bacteria were Firmicutes, Proteobacteria, Actinomycetes, and Bacteroides. This result is consistent with other bird studies, such as those investigating penguins, domestic geese (*Anser anser domesticus*), and neotropical birds [29,30,31,32]. During the 3 winter months, the relative abundance of Firmicutes, Proteobacteria, and Bacteroidetes did not change significantly, which indicates that the intestinal bacterial community of the hooded cranes was relatively stable. Firmicutes contribute to the breakdown of complex carbohydrates, polysaccharides, and fatty acids [33]. The relative abundance of Firmicutes in the intestine of hooded cranes may be related to its main plant source during the wintering period. ASV0360 *L. acidipiscis* and ASV0002 *L. acidipiscis* are the indicator species of the intestinal bacteria of hooded cranes in January and March, respectively. *L. acidipiscis* belongs to the dominant genus *Lactobacillus*, which is a probiotic that functions to improve food conversion [34]. As winter progresses, the plant resources of Shengjin Lake change, and the food resources available for hooded cranes decrease [13]. The increase in the relative abundance of *L. acidipiscis* increases the conversion rate of food. This shows the adaptability of the intestinal bacteria of hooded cranes for food reduction.

In this study, we found that the main foods of hooded cranes, namely, *O. sativa*, *P. criopolitanum*, and *Carex* spp., were all significantly correlated with the intestinal bacteria of hooded cranes and abundance of pathogenic bacteria. During the 3 winter months, the relative abundance of Actinobacteria in the gut of the hooded cranes showed an increasing trend. Actinobacteria are widespread in soil, water, and air [35], and their presence is usually associated with pathogens [36]. However, the ASV richness of pathogenic bacteria in hooded cranes did not differ significantly in these 3 months. Therefore, we considered that the increase in Actinobacteria may come from the environment.

The trend of the alpha diversity of the herbivorous food of hooded cranes was different from the alpha diversity of the gut bacteria. Although the main food species available to the hooded cranes changed, the plant nature of the food did not, which may explain why there was no obvious relationship between the change in intestinal bacterial alpha diversity and food composition. Beta diversity showed that the food composition and the intestinal bacteria of hooded cranes were extremely different in the 3 winter months. According to NMDS, we found that food composition and intestinal bacteria in each month all showed obvious aggregations. According to the two NMDS analyses, the aggregations in November and March were closer, which indicates that the food composition of the same habitat was more similar and the intestinal bacterial composition of hooded cranes eating food in the same habitat was also more similar. Because food-borne microbes can stay briefly in an animal’s gut [5], we speculate that the environmental microorganisms carried on food from the same habitat have few effects on the intestinal bacteria in hooded cranes, while the environmental microorganisms carried on foods from different habitats have a large effect on the intestinal bacteria of these birds.

## 5. Conclusions

Hooded cranes consume different types of food in the winter months in China, and they exhibit changes in intestinal microbial communities over this period. Our research shows that changes in diet greatly affect the bacterial diversity of hooded cranes, especially the main foods, *O. sativa*, *P. criopolitanum*, and *Carex* species. However, there are limitations to our study. Only 54 samples in 3 months were chosen for analysis, and there were only 15 replicates in March. In addition, plant nutrients affecting the intestinal bacterial community of hooded cranes, as well as environmental and endophytic microorganisms carried by plants, may be laterally imported into the intestines of hooded cranes and cause changes to the gut microbiome. Since we did not collect data on plant microorganisms, we were unable to provide direct evidence of the influence of foreign microorganisms on the intestinal bacterial community composition of hooded cranes. This should be made clear in future research.

## Figures and Tables

**Table 1:** Similarity percentage (SIMPER) analysis of the bacterial contribution to the difference for different months. ASV, amplicon sequence variant.

ASVs	Taxa	Contribution (%)		
		November vs. January	November vs. March	January vs. March
0360	*Lactobacillus acidipiscis*	21.65	-	22.93
0001	*Clostridium metallolevans*	11.23	11.92	-
0003	o__Bacillales	7.49	8.10	3.62
0005	g__*Paenibacillus*	3.30	3.28	-
0543	*Escherichia coli*	2.75	3.16	-
0002	*Lactobacillus acidipiscis*	-	12.76	13.25
0015	*Bacillus coahuilensis*	-	-	3.39
1329	*Lactobacillus acidipiscis*	-	-	2.18

Taxonomic abbreviations: o, order; g, genus.

**Table 2:** Foods related to the composition of the intestinal bacteria of hooded cranes according to Mantel test.

Foods	*R*	*p*-Value
*Oryza sativa*	0.448	0.001
*Polygonum criopolitanum*	0.364	0.001
*Carex* spp.	0.272	0.001
*Phalaris arundinacea*	0.241	0.001
*Lapsana apogonoides*	0.226	0.002
*Poa annua*	0.127	0.015
*Ranunculus japonicus*	0.121	0.024
*Potentilla supina*	0.116	0.033
*Vallisneria natans*	0.110	0.036

**Table 3:** Foods related to the composition of potential pathogenic bacteria in the faecal samples of hooded cranes according to Mantel test.

Foods	*R*	*p*-Value
*Oryza sativa*	0.293	0.001
*Phalaris arundinacea*	0.197	0.001
*Lapsana apogonoides*	0.180	0.015
*Polygonum criopolitanum*	0.177	0.001
*Carex* spp.	0.174	0.001
*Vallisneria natans*	0.143	0.033
*Alternanthera philoxeroides*	0.111	0.048

**Table 4:** The analysis of similarities (ANOSIM) test was used to compare differences in the food composition of hooded cranes.

Treatment	ANOSIM	
	*R*	*p*-Value
November vs. January	0.999	0.001
November vs. March	0.307	0.001
January vs. March	0.929	0.001

**Table 5:** The ANOSIM test was used to compare differences in the intestinal bacteria of hooded cranes.

Treatment	ANOSIM	
	*R*	*p*-Value
November vs. January	0.788	0.001
November vs. March	0.503	0.001
January vs. March	0.654	0.001

## Data Availability

The raw data have been submitted to the NCBI Sequence Read Archive (BioProject identifier (ID): PRJNA687921).

## Supplementary Materials

The following are available online at https://www.mdpi.com/2076-2615/11/2/433/s1: File S1, File S2, and File S3.

## References

[1] Kohl K.D. (2012). Diversity and function of the avian gut microbiota. J. Comp. Physiol. B.

[2] Li D., Wang P., Wang P., Hu X., Chen F. (2019). Targeting the gut microbiota by dietary nutrients: A new avenue for human health. Crit. Rev. Food Sci. Nutr..

[3] Zhu L., Wu Q., Dai J., Zhang S., Wei F. (2011). Evidence of cellulose metabolism by the giant panda gut microbiome. Proc. Natl. Acad. Sci. USA.

[4] Ley R.E., Hamady M., Lozupone C., Turnbaugh P.J., Ramey R.R., Bircher J.S., Schlegel M.L., Tucker T.A., Schrenzel M.D., Knight R. (2008). Evolution of mammals and their gut microbes. Science.

[5] David L.A., Maurice C.F., Carmody R.N., Gootenberg D.B., Button J.E., Wolfe B.E., Ling A.V., Devlin A.S., Varma Y., Fischbach M.A. (2014). Diet rapidly and reproducibly alters the human gut microbiome. Nat. Cell Biol..

[6] Roggenbuck M., Schnell I.B., Blom N., Bælum J., Bertelsen M.F., Sicheritz-Pontén T., Sørensen S.J., Gilbert M.T.P., Graves G.R., Hansen L.H. (2014). The microbiome of New World vultures. Nat. Commun..

[7] IUCN The IUCN Red List of Threatened Species 2016. Hooded Crane (*Grus monacha*).

[8] Song Y.W., Zhou L.Z. (2019). Effects of habitat changes on spatio-temporal pattern of the wintering waterbrid community at Shengjin Lake. J. Anhui Agric. Univ..

[9] Huang J., Guo Y.M. (2015). Diet of Hooded Crane (*Grus monacha*) in autumn, Lindian, China. Chin. J. Wildl..

[10] Hou J.J. (2019). Diet Niche Partitioning by Four Wintering Cranes in Poyang Lake. Master’s Thesis.

[11] Zhou B., Zhou L., Chen J., Cheng Y., Xu W. (2010). Diurnal time-activity budgets of wintering hooded cranes (*Grus monacha*) in Shengjin Lake, China. Waterbirds.

[12] Zhou L.L. (2013). Seasonal Shifts of Food Habit of the Hooded Crane (*Grus monacha*) Wintering in the Lakes of Yangtze River Floodplain in Anhui Province. Master’s Thesis.

[13] Wu L., Sun Y., Li J., Li Y., Wu Y., Li D. (2015). A phylogeny of the Passerida (Aves: Passeriformes) based on mitochondrial 12S ribosomal RNA gene. Avian Res..

[14] Knutie S.A., Gotanda K.M. (2018). A non-invasive method to collect fecal samples from wild birds for microbiome studies. Microb. Ecol..

[15] Zhang F., Xiang X., Dong Y., Yan S., Song Y., Zhou L. (2020). Significant differences in the gut bacterial communities of hooded crane (*Grus monacha*) in different seasons at a stopover site on the flyway. Animals.

[16] Bolyen E., Rideout J.R., Dillon M.R., Bokulich N.A., Abnet C.C., Al-Ghalith G.A., Alexander H., Alm E.J., Arumugam M., Asnicar F. (2019). Reproducible, interactive, scalable and extensible microbiome data science using QIIME 2. Nat. Biotechnol..

[17] Amir A., McDonald D., Navas-Molina J.A., Kopylova E., Morton J.T., Xu Z.Z., Kightley E.P., Thompson L.R., Hyde E.R., Gonzalez A. (2017). Deblur rapidly resolves single-nucleotide community sequence patterns. mSystems.

[18] Xiang X., Jin L., Yang Z., Zhang N., Zhang F. (2021). Dramatic shifts in intestinal fungal community between wintering hooded crane and domestic goose. Avian Res..

[19] Anderson M.J., Walsh D.C.I. (2013). PERMANOVA, ANOSIM, and the Mantel test in the face of heterogeneous dispersions: What null hypothesis are you testing?. Ecol. Monogr..

[20] Gibert C., Escarguel G. (2018). PER-SIMPER-A new tool for inferring community assembly processes from taxon occurrences. Glob. Ecol. Biogeogr..

[21] Segata N., Izard J., Waldron L., Gevers D., Miropolsky L., Garrett W.S., Huttenhower C. (2011). Metagenomic biomarker discovery and explanation. Genome Biol..

[22] Zhao G., Zhou L., Dong Y., Cheng Y., Song Y. (2017). The gut microbiome of hooded cranes (*Grus monacha*) wintering at Shengjin Lake, China. Microbiology.

[23] Wan W., Zhou L., Song Y. (2016). Shifts in foraging behavior of wintering hooded cranes (*Grus monacha*) in three different habitats at Shengjin Lake, China. Avian Res..

[24] Wei Z., Zheng M., Zhou L., Xu W. (2020). Flexible foraging response of wintering hooded cranes (*Grus monacha*) to food availability in the lakes of the Yangtze River floodplain, China. Animals.

[25] Semova I., Carten J.D., Stombaugh J., Mackey L.C., Knight R., Farber S.A., Rawls J.F. (2012). Microbiota regulate intestinal absorption and metabolism of fatty acids in the zebrafish. Cell Host Microbe.

[26] Ma S.Y. (2011). Study on Vegetation Community Characteristics in Wetland of Shengjin Lake in Anhui Province. Master’s Thesis.

[27] Zou H.F., Feng X.D., Wu Q.M., Wu Y.N., Hao M., Ma J.Z. (2012). Diet component and preference of white-naped crane during courtship period in Zhalong Nature Reserve. J. Northeast For. Univ..

[28] Jiao S.W., Jiang K.Y., Zuo A.J., Wu M., Lei G.C., Zhou Y. (2017). Foraging behavior and food resources of wintering *Grus monacha* in China. Sichuan Dong Wu.

[29] Barbosa A., Balagué V., Valera F., Martínez A., Benzal J., Motas M., Diaz J.I., Mira A., Pedrós-Alió C. (2016). Age-related differences in the gastrointestinal microbiota of chinstrap penguins (*Pygoscelis antarctica*). PLoS ONE.

[30] Dewar M.L., Arnould J.P.Y., Dann P., Trathan P., Groscolas R., Smith S. (2013). Interspecific variations in the gastrointestinal microbiota in penguins. Microbiology.

[31] Fu R., Xiang X., Dong Y., Cheng L., Zhou L. (2020). Comparing the intestinal bacterial communities of sympatric wintering hooded crane (*Grus monacha*) and domestic goose (*Anser anser domesticus*). Avian Res..

[32] Hird S.M., Sanchez C., Carstens B.C., Brumfield R.T. (2015). Comparative gut microbiota of 59 neotropical bird species. Front. Microbiol..

[33] Flint H.J., Bayer E.A., Rincon M.T., Lamed R., White B.A. (2008). Polysaccharide utilization by gut bacteria: Potential for new insights from genomic analysis. Nat. Rev. Genet..

[34] Altaher Y., Jahromi M., Ebrahim R., Zulkifli I., Liang J.B. (2015). *Lactobacillus Pentosus* Ita23 and *L. Acidipiscis* Ita44 enhance feed conversion efficiency and beneficial gut microbiota in broiler chickens. Braz. J. Poult. Sci..

[35] Mikaelyan A., Dietrich C., Köhler T., Poulsen M., Sillam-Dussès D., Brune A. (2015). Diet is the primary determinant of bacterial community structure in the guts of higher termites. Mol. Ecol..

[36] Santos S.S., Pardal S., Proença D.N., Lopes R.J., Ramos J.A., Mendes L., Morais P.V. (2012). Diversity of cloacal microbial community in migratory shorebirds that use the Tagus estuary as stopover habitat and their potential to harbor and disperse pathogenic microorganisms. FEMS Microbiol. Ecol..

