# Supplementary material for: Effects of Food Changes on Intestinal Bacterial Diversity of Wintering Hooded Cranes (Grus monacha)
Source: Animals (Basel). 2021 Feb 7;11(2):433. doi: 10.3390/ani11020433 (PMC7915383; doi:10.3390/ani11020433)
Supplement: Supplementary file 1 [file animals-11-00433-s001.zip › animals-1079179-supplementary-S1.pdf]

File S1: Supplementary materials

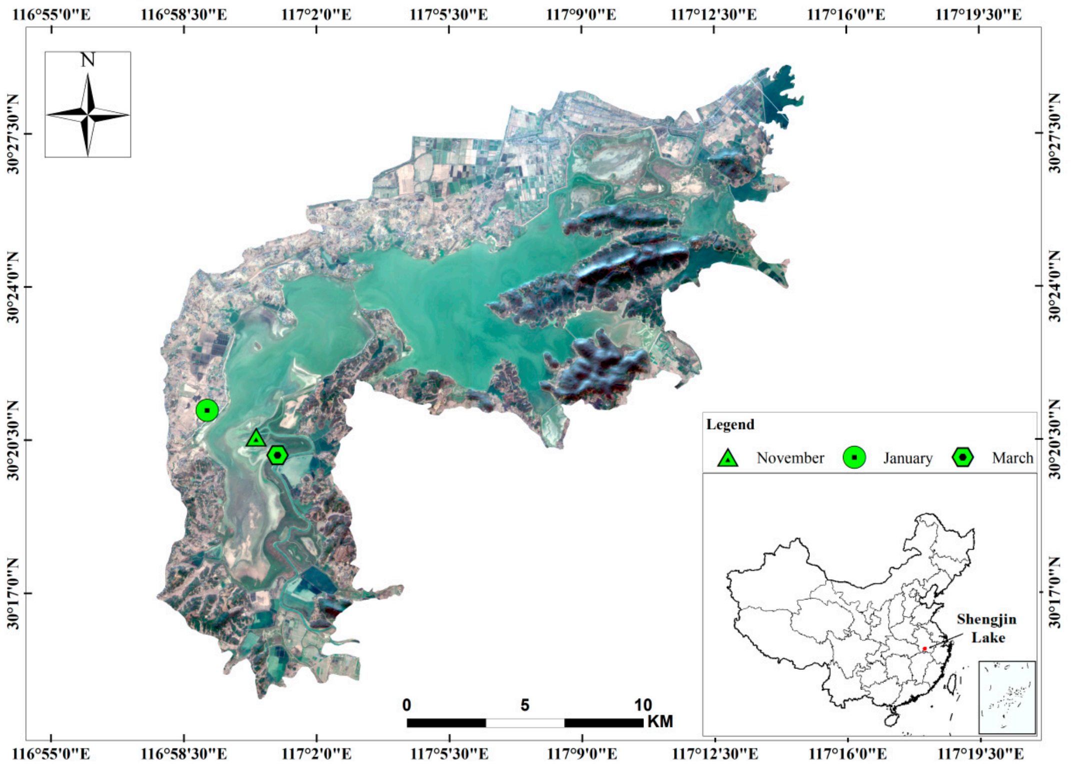

Figure S1. Faecal sampling sites of hooded cranes at Shengjin Lake.

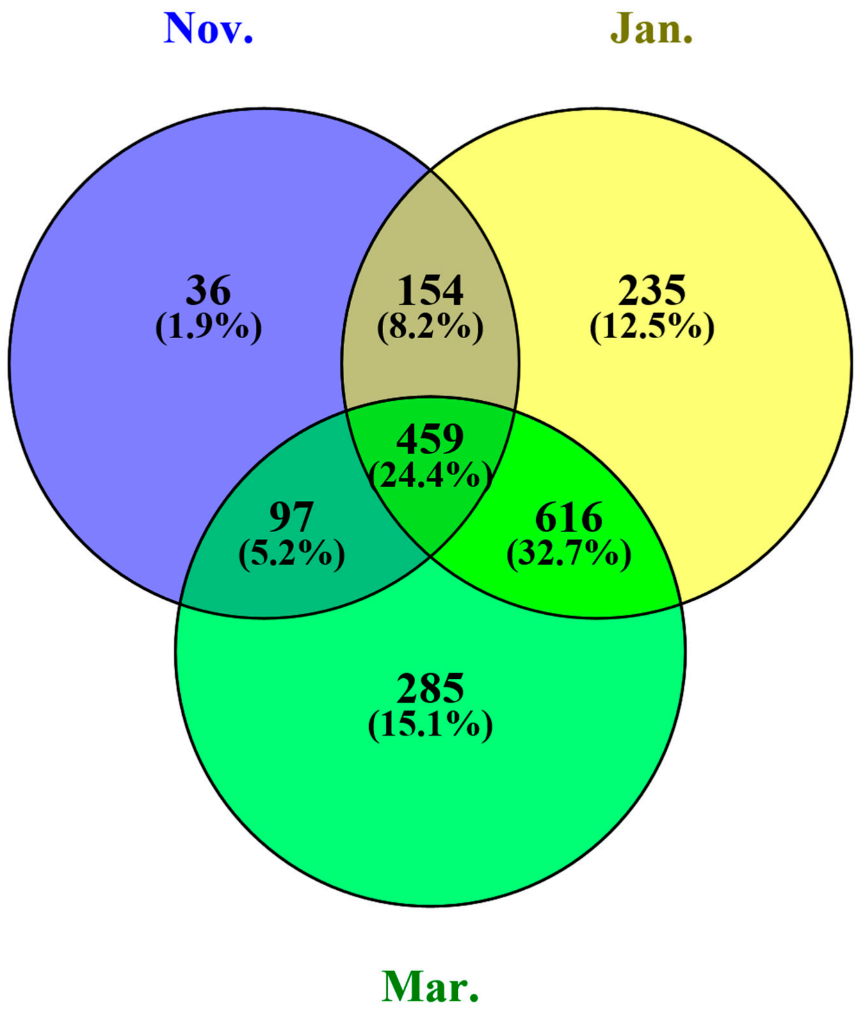

Figure S2. The overlapping of intestinal bacterial amplicon sequence variants (ASVs) in hooded cranes among the three months.

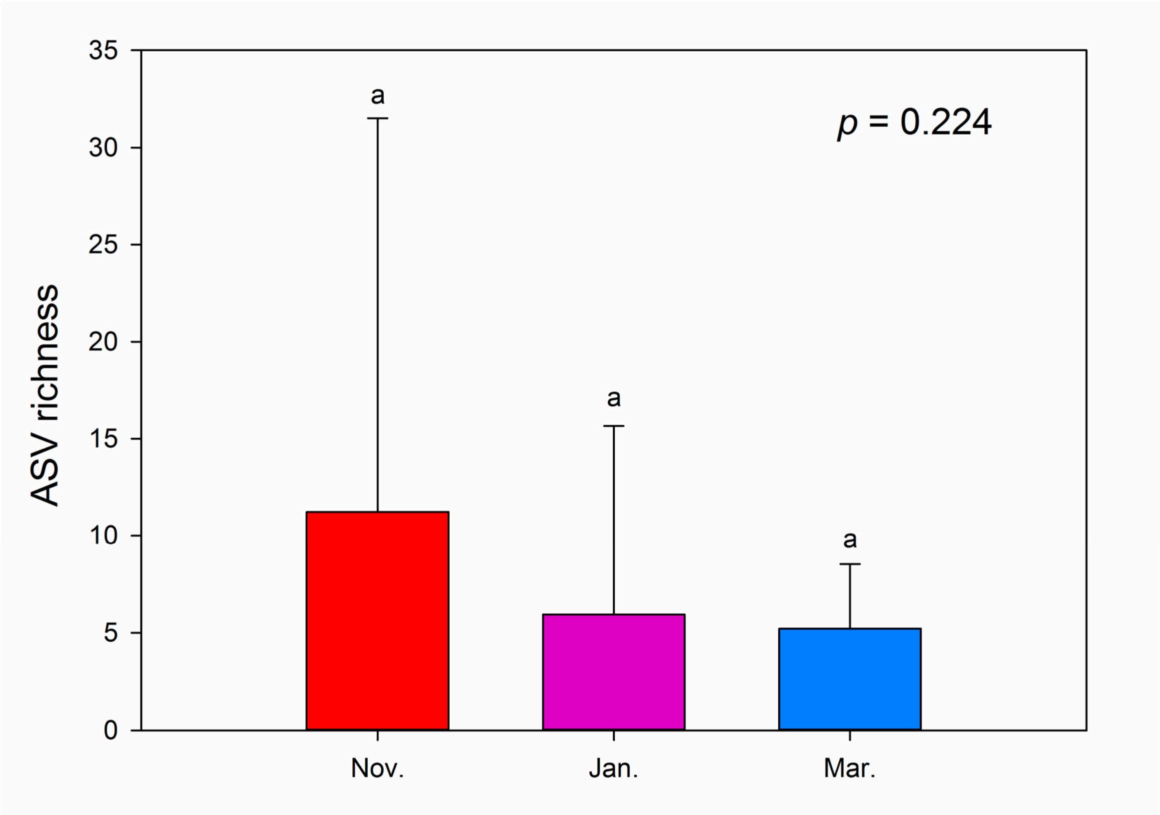

Figure S3. The amplicon sequence variants (ASV) richness of intestinal pathogenic bacteria in hooded cranes in the three months. Letters over the bars indicate pair-wise differences based on Dunn-Bonferroni test at $p < 0.05$ level. Coloured bars indicate mean value, and error bars represent standard deviation.

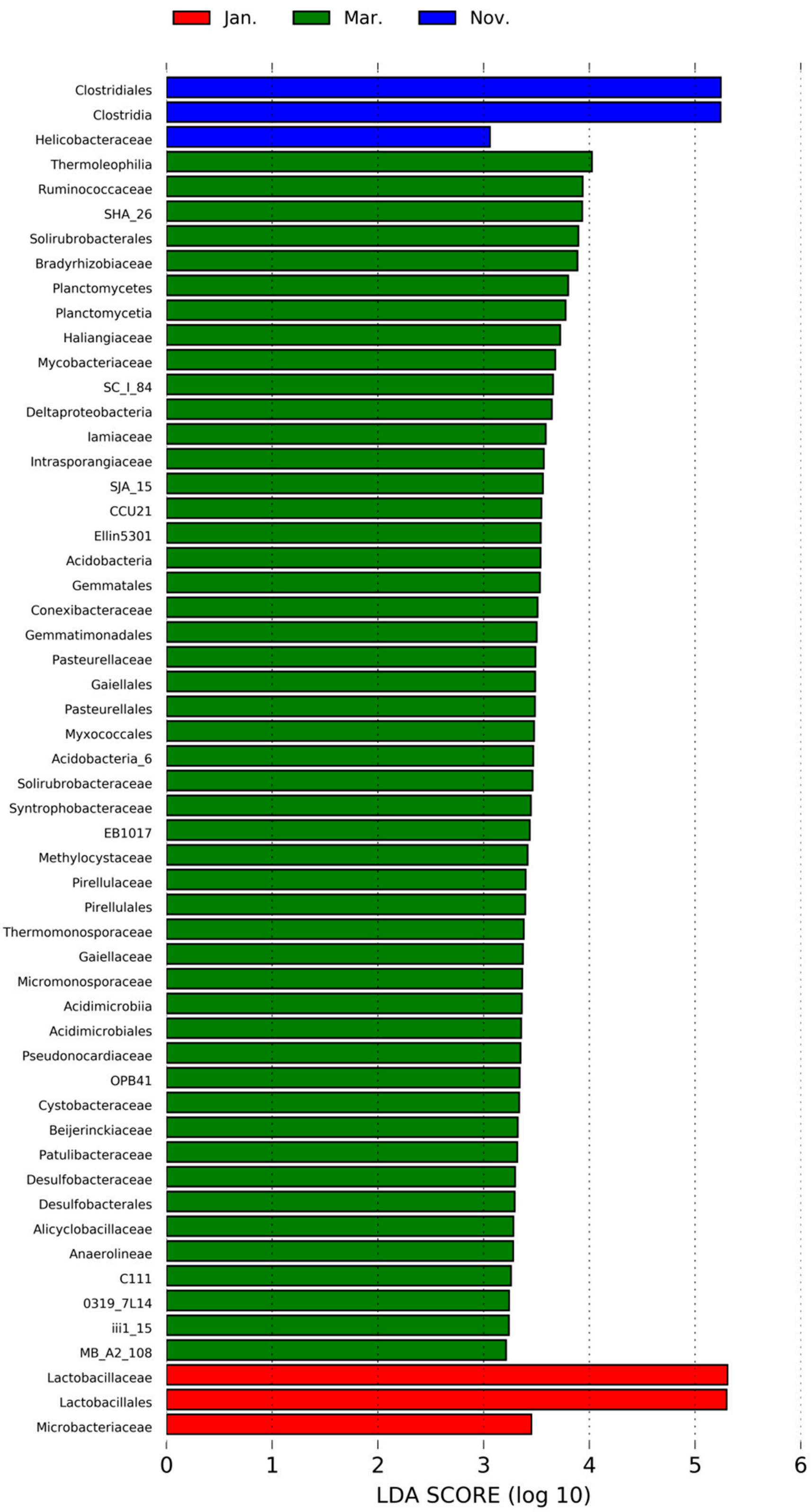

Figure S4. Identified phylotype biomarkers ranked by effect size in hooded cranes. Phylotype biomarkers were identified as being significantly abundant when samples from each month were compared and the alpha value was < 0.05.

Table S1. The distribution of data about the faecal samples of hooded cranes analyzed using the Kolmogorov-Smirnov test.

**Items**	**Kolmogorov-Smirnov Test (*p*-Value)**	**Distribution**
Bacterial Chao1	0.427	Normal
Bacterial ASV richness	0.920	Normal
Pathogenic ASV richness	<0.001	Non-normal
Simpson of available food	0.894	Normal
Shannon of available food	0.856	Normal
Firmicutes	0.313	Normal
Proteobacteria	0.144	Normal
Actinobacteria	0.208	Normal
Bacteroidetes	<0.001	Non-normal
Lactobacillus	0.113	Normal
Clostridium (f__Peptostreptococcaceae)	<0.001	Non-normal
Paenibacillaceae	0.022	Non-normal
Clostridium (f__Clostridiaceae)	<0.001	Non-normal
Bacillus	0.001	Non-normal
Methylobacterium	<0.001	Non-normal
Martelella	<0.001	Non-normal
Enterococcus	<0.001	Non-normal
Escherichia	<0.001	Non-normal
Arthrobacter	<0.001	Non-normal
Nocardioides	0.014	Non-normal
Epulopiscium	<0.001	Non-normal

Normal distribution: $p > 0.05$; Non-normal distribution: $p \leq 0.05$. Taxonomic abbreviations: f, family.

Table S2. The relative density of the available foods of hooded cranes.

Scientifc Name	RD ± SD in Winter (%)	RD ± SD in Nov. (%)	RD ± SD in Jan. (%)	RD ± SD in Mar. (%)
Polygonum criopolitanum	33.33 ± 15.09	47.13 ± 9.01	20.66 ± 5.54	32.76 ± 15.09
Carex spp.	21.16 ± 13.75	29.36 ± 9.74	7.87 ± 6.08	28.48 ± 11.94
Oryza sativa	19.27 ± 21.61	3.99 ± 3.20	46.49 ± 7.00	2.32 ± 2.15
Phalaris arundinacea	6.96 ± 3.64	9.35 ± 2.91	4.48 ± 2.39	7.24 ± 3.87
Poa annua	3.99 ± 5.12	1.26 ± 1.14	5.89 ± 3.76	4.91 ± 7.88
Potentilla supina	3.35 ± 7.45	0.39 ± 0.58	1.30 ± 1.21	9.84 ± 12.03
Artemisia selengensis	3.27 ± 3.91	3.10 ± 2.31	0.65 ± 0.87	6.97 ± 5.06
Ranunculus japonicus	1.80 ± 4.17	0.00 ± 0.00	0.82 ± 0.97	5.38 ± 6.70
Vallisneria natans	1.12 ± 2.13	3.07 ± 2.68	0.13 ± 0.31	0.00 ± 0.00
Triticum aestivum	0.99 ± 2.53	0.00 ± 0.00	2.65 ± 3.64	0.03 ± 0.12
Lapsana apogonoides	0.94 ± 1.53	0.09 ± 0.28	2.36 ± 1.73	0.13 ± 0.27
Alternanthera philoxeroides	0.69 ± 1.22	0.37 ± 0.67	1.52 ± 1.59	0.00 ± 0.00
Rumex dentatus	0.68 ± 1.12	0.12 ± 0.40	1.21 ± 1.28	0.66 ± 1.21
Alopecurus aequalis	0.47 ± 1.08	0.17 ± 0.43	1.06 ± 1.56	0.08 ± 0.21
Ceratophyllum demersum	0.46 ± 0.75	0.43 ± 0.74	0.70 ± 0.93	0.17 ± 0.30
Brassica campestris	0.43 ± 0.96	0.00 ± 0.00	1.11 ± 1.33	0.06 ± 0.22
Unknown species	0.33 ± 0.58	0.34 ± 0.55	0.45 ± 0.75	0.14 ± 0.29
Zizania latifolia	0.32 ± 0.63	0.48 ± 0.84	0.31 ± 0.48	0.12 ± 0.47
Medicago spp.	0.32 ± 0.64	0.21 ± 0.45	0.19 ± 0.43	0.62 ± 0.96
Spirogyra communis	0.07 ± 0.21	0.04 ± 0.16	0.07 ± 0.22	0.09 ± 0.25
Setaria viridis	0.03 ± 0.16	0.05 ± 0.20	0.04 ± 0.17	0.00 ± 0.00
Potamogeton malaianus	0.03 ± 0.15	0.04 ± 0.17	0.04 ± 0.18	0.00 ± 0.00
Kalimeris indica	0.01 ± 0.10	0.04 ± 0.17	0.00 ± 0.00	0.00 ± 0.00

RD, relative density; SD, standard deviation.

Table S3. Sequencing and amplicon sequence variants (ASVs) classification information in the faecal samples of hooded cranes.

Sample	Sequences	ASVs	Phylum	Class	Order	Family	Genus
Nov.1	11559	99	6	12	19	40	54
Nov.2	8229	111	5	12	21	45	60
Nov.3	5323	87	6	11	16	30	41
Nov.4	10810	77	4	8	14	26	38
Nov.5	14774	142	5	12	21	53	78
Nov.6	14955	106	6	12	19	38	55
Nov.7	12365	168	5	12	22	57	86
Nov.8	5768	40	3	7	11	17	22
Nov.9	5829	211	9	21	38	66	89
Nov.10	5226	158	7	17	25	56	78
Nov.11	10799	126	5	11	19	45	64
Nov.12	13458	102	5	11	20	39	52
Nov.13	10841	62	4	7	10	23	31
Nov.14	9100	459	10	25	55	112	175
Nov.15	14579	108	7	13	22	42	54
Nov.16	11408	143	6	11	21	46	65
Nov.17	10710	58	5	9	12	21	30
Nov.18	9676	140	7	15	26	53	70
Nov.19	11140	96	6	12	20	40	45
Jan.1	17725	427	13	38	72	124	173
Jan.2	18389	291	10	27	51	101	142
Jan.3	22780	131	4	11	20	46	66
Jan.4	23333	221	10	19	34	76	108
Jan.5	7588	147	7	17	27	58	76
Jan.6	10060	247	9	20	33	75	110
Jan.7	22503	226	10	25	41	78	114
Jan.8	24947	175	9	22	36	70	99
Jan.9	21440	144	6	12	23	52	74

Jan.10	10410	541	12	40	77	144	215
Jan.11	17159	261	8	25	40	85	134
Jan.12	17415	352	10	29	59	110	158
Jan.13	16174	479	13	36	74	133	191
Jan.14	13407	621	13	36	75	144	220
Jan.15	9708	169	6	12	22	54	78
Jan.16	7672	265	7	18	35	79	120
Jan.17	18320	244	7	18	34	79	119
Jan.18	21173	182	5	14	22	60	93
Jan.19	22471	52	3	6	10	24	31
Jan.20	23952	102	4	9	17	39	61
Mar.1	6754	517	10	34	61	125	195
Mar.2	11692	168	7	18	27	59	84
Mar.3	9465	68	5	11	17	37	42
Mar.4	6706	264	8	23	35	85	128
Mar.5	16963	550	12	31	70	134	207
Mar.6	12240	314	10	26	51	101	141
Mar.7	11020	521	11	32	67	137	211
Mar.8	12414	483	13	32	61	118	178
Mar.9	7411	652	13	36	76	138	208
Mar.10	6330	358	7	23	49	101	152
Mar.11	6513	329	10	27	47	97	140
Mar.12	15234	618	13	40	79	150	225
Mar.13	16673	339	10	29	52	107	155
Mar.14	10056	312	9	22	38	89	127
Mar.15	10822	239	7	19	38	79	112

Table S4. Relative abundance of the dominant phyla in the faecal samples of hooded cranes in the three months.

Phylum	Distribution	p-Value	Relative abundance (%)		
			Nov.	Jan.	Mar.
Firmicutes	Normal	0.287	75.8 (27.6) [a]	65.1 (21.1) [a]	64.9 (21.7) [a]
Proteobacteria	Normal	0.843	19.6 (25.2) [a]	20.3 (12.4) [a]	16.9 (10.7) [a]
Actinobacteria	Normal	<0.001	3.13 (4.95) [b]	10.0 (7.59) [a]	12.8 (7.18) [a]
Bacteroidetes	Non-normal	0.265	0.151 (0.194) [a]	2.27 (6.94) [a]	2.50 (5.81) [a]

Letters of "a" and "b" indicate pair-wise differences based on Tukey HSD test and Dunn-Bonferroni test at $p < 0.05$ level.

Table S5. Relative abundance of the dominant bacterial genus in the faecal samples of hooded cranes in the three months.

Genus	Distribution	p-Value	Relative abundance (%)		
			Nov.	Jan.	Mar.
Lactobacillus (f__Lactobacillaceae)	Normal	<0.001	4.40 (5.87) [b]	45.6 (27.7) [a]	31.0 (23.6) [a]
Clostridium (f__Peptostreptococcaceae)	Non-normal	<0.001	22.1 (19.4) [a]	1.20 (2.39) [b]	3.09 (5.46) [b]
Paenibacillus (f__Paenibacillaceae)	Non-normal	0.199	6.85 (8.33) [a]	4.00 (5.52) [a]	5.75 (3.86) [a]
Clostridium (f__Clostridiaceae)	Non-normal	<0.001	12.6 (13.5) [a]	1.01 (1.08) [b]	1.70 (2.85) [b]
Bacillus (f__Bacillaceae)	Non-normal	0.031	4.39 (4.30) [ab]	5.16 (10.35) [b]	5.70 (5.40) [a]
Methylobacterium (f__Methylobacteriaceae)	Non-normal	<0.001	6.06 (10.6) [a]	1.40 (1.57) [b]	0.836 (0.895) [b]
Martelella (f__Aurantimonadaceae)	Non-normal	<0.001	1.72 (1.71) [a]	3.68 (6.98) [a]	0.110 (0.146) [b]
Enterococcus (f__Enterococcaceae)	Non-normal	0.635	2.07 (6.17) [a]	2.91 (9.35) [a]	0.413 (0.658) [a]
Escherichia (f__Enterobacteriaceae)	Non-normal	0.069	5.11 (20.4) [a]	0.0172 (0.0277) [a]	0.327 (1.22) [a]
Arthrobacter (f__Micrococcaceae)	Non-normal	0.006	0.480 (0.889) [b]	2.60 (5.45) [ab]	2.41 (4.03) [a]
Nocardioides (f__Nocardioidaceae)	Non-normal	0.003	0.687 (0.889) [b]	1.28 (1.50) [ab]	2.71 (2.00) [a]
Epulopiscium (f__Lachnospiraceae)	Non-normal	<0.001	3.36 (3.53) [a]	0.117 (0.179) [b]	0.893 (0.112) [b]

Letters of "a" and "b" indicate pair-wise differences based on Tukey HSD test and Dunn-Bonferroni test at $p < 0.05$ level.
Taxonomic abbreviations: f, family.

Table S6. The identified potential pathogens carried by hooded cranes.

Potential Pathogens	Distribution	*p*-Value	Relative abundance (%)		
			Nov.	Jan.	Mar.
Escherichia coli	Non-normal	0.069	5.11 (20.4) [a]	0.0172 (0.0277) [a]	0.327 (1.22) [a]
Clostridium botulinum	Non-normal	<0.001	2.62 (4.51) [a]	0.204 (0.362) [b]	0.528 (1.01)[b]
Enterococcus casseliflavus	Non-normal	0.062	0.765 (0.266) [a]	2.62 (9.39) [a]	0.0038 (0.0107)[a]
Enterococcus cecorum	Non-normal	<0.001	1.53 (4.54) [ab]	0.105 (0.471) [c]	0.410 (0.656) [b]
Streptococcus equi	Non-normal	<0.001	0.106 (0.244) [b]	0 (0)[c]	1.86 (2.67) [a]
Mycobacterium llatzerense	Non-normal	<0.001	0.0393 (0.0709) [b]	0.382 (0.599) [a]	0.799 (0.616) [a]
Rhodococcus globerulus	Non-normal	0.002	0.0554 (0.149) [b]	0.0751 (1.26) [a]	0.107 (0.154) [ab]
Streptococcus alactolyticus	Non-normal	0.036	0.200 (0.690) [b]	0.101 (0.202) [ab]	0.570 (1.18) [a]
Atopobium rimae	Non-normal	0.427	0 (0) [a]	0.690 (3.08) [a]	0 (0) [a]
Aurantimonas altamirensis	Non-normal	0.560	0.115 (0.226) [a]	0.430 (0.753) [a]	0.0383 (0.0307) [a]
Clostridium difficile	Non-normal	<0.001	0.427 (0.588) [a]	0.0067 (0.0258) [b]	0.0434 (0.116) [b]
Ewingella americana	Non-normal	0.333	0.369 (1.21) [a]	0.0067 (0.0179) [a]	0 (0) [a]
Paenibacillus amylolyticus	Non-normal	0.033	0.0020 (0.00603) [ab]	0.341 (1.35) [a]	0 (0) [b]
Streptomyces reticuliscabiei	Non-normal	0.385	0.0816 (0.224) [a]	0.110 (0.221) [a]	0.0549 (0.0511) [a]
Helicobacter pullorum	Non-normal	<0.001	0.149 (0.179) [a]	0 (0) [b]	0.112 (0.276) [b]
Rhodococcus equi	Non-normal	0.018	0.0735 (0.320) [a]	0.0201 (0.0432) [ab]	0.0242(0.0302) [b]
Clostridium bifermentans	Non-normal	0.857	0.0534 (0.170) [a]	0.0364 (0.111) [a]	0.0089 (0.0203) [a]
Rhodococcus fascians	Non-normal	0.002	0.0675 (0.0742) [a]	0.0172 (0.0402) [b]	0.0344 (0.0584) [b]
Kerstersia gyiorum	Non-normal	0.048	0.0070 (0.0307) [a]	0.0316 (0.0505) [a]	0.0702 (0.235) [a]
Streptobacillus moniliformis	Non-normal	0.006	0.0383 (0.0868) [ab]	0 (0) [b]	0.0434 (0.0598) [a]
Clostridium sordellii	Non-normal	0.535	0.0534 (0.173) [a]	0.00478 (0.0214) [a]	0.00510 (0.0135) [a]

Clostridium neonatale	Non-normal	0.296	0.00302 (0.00960) [a]	0.0268 (0.0870) [a]	0 (0) [a]
Campylobacter rectus	Non-normal	0.022	0.00705 (0.0146) [ab]	0 (0) [b]	0.0268 (0.0526) [a]
Lactococcus garvieae	Non-normal	0.018	0 (0) [b]	0.00191 (0.00856) [ab]	0.0255 (0.0504) [a]
Acinetobacter guillouiae	Non-normal	0.552	0 (0) [a]	0.000957 (0.00428) [a]	0.0268 (0.104) [a]
Agromyces mediolanus	Non-normal	0.009	0.00705 (0.0146) [ab]	0 (0) [b]	0.0166 (0.0227) [a]
Robinsoniella peoriensis	Non-normal	0.019	0.00101 (0.00439) [ab]	0 (0) [b]	0.0242 (0.0534) [a]
Vagococcus salmoninarum	Non-normal	0.398	0.0201 (0.0878) [a]	0 (0) [a]	0 (0) [a]
Clostridium subterminale	Non-normal	0.034	0.0101 (0.0184) [a]	0 (0) [b]	0.0102 (0.0395) [ab]
Nocardia concava	Non-normal	0.057	0 (0) [a]	0.0124 (0.0293) [a]	0.00638 (0.118) [a]
Mycobacterium arupense	Non-normal	0.802	0.00604 (0.0192) [a]	0.00287 (0.00701) [a]	0.00510 (0.0114) [a]
Elizabethkingia meningoseptica	Non-normal	0.273	0 (0) [a]	0 (0) [a]	0.0166 (0.0642) [a]
Burkholderia gladioli	Non-normal	0.231	0 (0) [a]	0.00287 (0.00701) [a]	0.00765 (0.0214) [a]
Corynebacterium simulans	Non-normal	0.017	0 (0) [b]	0 (0) [b]	0.0102 (0.0238) [a]
Paenibacillus larvae	Non-normal	0.090	0 (0) [a]	0.00574 (0.0126) [a]	0.00128 (0.00494) [a]
Staphylococcus saprophyticus	Non-normal	0.177	0 (0) [a]	0.00574 (0.0177) [a]	0 (0) [a]
Acinetobacter lwoffii	Non-normal	0.273	0 (0) [a]	0 (0) [a]	0.00765 (0.0296) [a]
Kurthia gibsonii	Non-normal	0.552	0 (0) [a]	0.000957 (0.00428) [a]	0.00383 (0.0148) [a]
Sphingobacterium multivorum	Non-normal	0.071	0 (0) [a]	0.00383 (0.0100) [a]	0 (0) [a]

Letters of "a", "b" and "c" indicate pair-wise differences based on Dunn-Bonferroni test at $p < 0.05$ level.

Table S7. Indicator species in the faecal samples of hooded cranes in the three months.

Treatment	Indicator Bacteria	Indicator Value	*p*-Value	Relative Abundance (%)	Taxa
Nov.	ASV0001	0.850	0.001	8.676	*Clostridium metallolevans*
	ASV0003	0.611	0.008	7.339	o__Bacillales
	ASV0005	0.604	0.005	3.613	g__*Paenibacillus*
	ASV0006	0.608	0.009	0.583	f__Planococcaceae
	ASV0007	0.480	0.035	1.789	*Bacillus coahuilensis*
	ASV0008	0.775	0.003	0.658	g__*Turicibacter*
	ASV0011	0.791	0.001	0.540	g__*Clostridium*
	ASV0017	0.972	0.001	1.710	*Clostridium celatum*
	ASV0034	0.883	0.001	0.943	g__*Clostridium*
	ASV0045	0.899	0.001	1.936	*Methylobacterium adhaesivum*
	ASV0054	0.876	0.001	0.845	g__*Epulopiscium*
	ASV0071	0.665	0.002	0.652	f__mitochondria
Jan.	ASV0360	0.840	0.001	16.823	*Lactobacillus acidipiscis*
	ASV0376	0.779	0.001	1.443	g__*Martelella*
	ASV1329	0.500	0.001	1.420	*Lactobacillus acidipiscis*
Mar.	ASV0002	0.839	0.001	7.433	*Lactobacillus acidipiscis*
	ASV0004	0.884	0.001	0.533	*Streptococcus equi*
	ASV0013	0.560	0.05	0.625	g__*Paenisporosarcina*
	ASV0019	0.699	0.001	0.528	g__*Paenibacillus*
	ASV0020	0.731	0.001	0.563	g__*Paenibacillus*
	ASV0023	0.654	0.001	0.543	*Lactobacillus acidipiscis*

Taxonomic abbreviations: o, order; f, family; g, genus.
