# Supplementary material for: Effects of Food Changes on Intestinal Bacterial Diversity of Wintering Hooded Cranes (Grus monacha)
Source: Animals (Basel). 2021 Feb 7;11(2):433. doi: 10.3390/ani11020433 (PMC7915383; doi:10.3390/ani11020433)
Supplement: Supplementary file 1 [file animals-11-00433-s001.zip › animals-1079179-supplementary-S2.pdf]

File S2: Supplementary Materials

Supporting Materials and Methods

Faecal DNA Extraction

QIAamp Fast DNA Stool Mini Kits were used to extract faecal DNA, quantified with a NanoDrop ND-1000 (Thermo Scientific, United States), and stored at −20 °C until subsequent analysis.

Species Identification

The COI barcode area was amplified to confirm whether the DNA samples belonged to hooded cranes. We used primers F1 (TTCTCCAACCACAAAGACATTGGCAC) and R1 (ACGTGGGAGATAATTCCAAATCCTG) to identify hooded cranes [1]. A reaction mixture of 50 μL was used, comprising 25 μL of SuperMix (2× Easy Taq® PCR SuperMix (+ dye), TRANSGEN), 1 μL of DNA template, 1 μL of forward primer (10 μM), 1 μL of reverse primer (10 μM), and 22 μL of nuclease-free water. A PCR Amplifier was used for DNA amplification. The cycling parameters were as follows: 5 min at 95 °C for initial denaturation; 30 s at 95 °C for degeneration, 45 s at 55 °C for annealing, 90 s at 72 °C for extension, and 35 cycles; 10 min at 72 °C for the final extension. The final product was subjected to gel electrophoresis, and samples with clear strips visualised using an ultraviolet analyser were sent to Sangon Biotech in Shanghai for sequencing. PCR products were sequenced and subjected to BLAST analysis (>97% sequence identity) at the National Center for Biotechnology Information (NCBI).

Amplicon Library Preparation

The purified DNA (50 ng) was isolated from each sample and amplified as a template. The primer set F515/R907 with a sequencing adapter and unique identifier label was used to amplify the V4–V5 variant region of the bacterial 16S rRNA gene fragment using the Illumina Mi-Seq platform (PE 250) [2] of Majorbio (Shanghai, China). PCR was performed in a 50 μL reaction mixture which comprised deoxynucleoside triphosphate (200 mM), forward and reverse primer (0.4 mM), and Taq DNA polymerase (2 U). The PCR parameters were as follows: denaturation at 94 °C for 45 s, 35 cycles of annealing at 55 °C for 45 s, extension at 72 °C for 45 s, and a final extension at 72 °C for 10 min. To detect contamination, we included negative controls (no template). The negative controls contained no detectable PCR products. Triplicate reaction mixtures of each sample were pooled and purified using an agarose gel DNA purification kit prior to sequencing. Identical amounts of PCR product (10 pg) were used to sequence each sample.

References

1. Saitoh, T.; Sugita, N.; Someya, S.; Iwami, Y.; Kobayashi, S.; Kamigaich, H.; Higuch, A.; Asai, S.; Yamamoto, Y.; Nishiumi, I. DNA barcoding reveals 24 distinct lineages as cryptic bird species candidates in and around the Japanese Archipelago. *Mol. Ecol. Resour.* **2014**, *15*, 177–186. doi: 10.1111/1755-0998.12282

2. Biddle, J.F.; Fitz-Gibbon, S.; Schuster, S.C.; Brenchley, J.E.; House, C.H. Metagenomic signatures of the Peru margin subsea floor biosphere show a genetically distinct environment. *Proc. Natl. Acad. Sci. U.S.A.* **2008**, *105*, 10583–10588. doi: 10.1073/pnas.0709942105
