# Supplementary material for: Effects of Food Changes on Intestinal Bacterial Diversity of Wintering Hooded Cranes (Grus monacha)
Source: Animals (Basel). 2021 Feb 7;11(2):433. doi: 10.3390/ani11020433 (PMC7915383; doi:10.3390/ani11020433)
Supplement: Supplementary file 1 [file animals-11-00433-s001.zip › animals-1079179-supplementary-S3.pdf]

File S3: Supplementary materials

Table S1. The identified potential pathogens carried by hooded cranes in this study

Pathogenic Species	Symptom	Infected Target
Escherichia coli	Meningoencephalitis	Humans, Animals [1]
Clostridium botulinum	Infant poisoning, Death or debilitation in waterfowls	Humans [2], Birds [3]
Enterococcus casseliflavus	Endocarditis, Endometritis	Horses [4,5]
Enterococcus cecorum	Sepsis and ventriculitis in humans, Arthritis and osteomyelitis in chickens	Humans, Poultry [6]
Streptococcus equi	Pneumonia, Septicemia, Meningitis	Humans, Horses, Dogs, Pigs, etc [7]
Mycobacterium llatzerense	Abdominal abscess, Lung infection	Humans [8,9]
Rhodococcus globerulus	Bacteremia	Humans [10]
Streptococcus alactolyticus	Pneumonia	Humans [11]
Atopobium rimae	Bacteremia	Humans [12]
Aurantimonas altamirensis	Bloodstream infection	Humans [13]
Clostridium difficile	Infectious diarrhea	Humans [14]
Ewingella americana	Bacterial brown rot disease, Pneumonia	Needle mushroom [15], Humans [16]
Paenibacillus amylolyticus	Bacteremia	Humans [17]
Streptomyces reticuliscabiei	Netted scab	Potato [18]
Helicobacter pullorum	Gastroenteritis, Colitis and hepatitis	Poultry, Humans [19]
Rhodococcus equi	Infective endocarditis, Suppurative pneumonia	Humans [20], Foals [21]
Clostridium bifermentans	Necrotizing endometritis, Vine decline	Humans [22], Kiwifruit [23]
Rhodococcus fascians	Leafy gall syndrome	Dicotyledonous herbs [24]
Kerstersia gyiorum	Chronic suppurative otitis media, Bacteremia	Humans [25,26]
Streptobacillus moniliformis	Spinal epidural abscess, Rat bite fever	Humans [27,28]
Clostridium sordellii	Hemorrhagic and necrotizing gastroenteropathy, Bacteremia	Dogs [29], Humans [30]
Clostridium neonatale	Neonatal diarrhoea	Pigs [31]
Campylobacter rectus	Fatal thoracic empyema, Acute otitis media	Humans [32,33]
Lactococcus garvieae	Lactococcosis	Fish [34]
Acinetobacter guillouiae	Septic shock	Humans [35]
Agromyces mediolanus	Bacteremia	Humans [36]
Robinsoniella peoriensis	Bacteremia	Humans [37]

Vagococcus salmoninarum	Coldwater streptococcosis	Rainbow trout [38]
Clostridium subterminale	Vine decline, Pleuropulmonary infection	Kiwifruit, Humans [39]
Nocardia concava	Systemic nocardiosis, Pulmonary nocardiosis	Humans [40,41]
Mycobacterium arupense	Finger osteomyelitis, Tenosynovitis	Humans [42,43]
Elizabethkingia meningoseptica	Bacteremia, Meningitis	Humans [44,45]
Burkholderia gladioli	Panicle blight and grain discoloration, Bacterial brown spot	Rice [46], *Phalaenopsis* spp. [47]
Corynebacterium simulans	Pyogenic spondylitis	Humans [48]
Paenibacillus larvae	American foulbrood	Honey bees [49]
Staphylococcus saprophyticus	Uncomplicated lower urinary tract infections	Humans [50]
Acinetobacter lwoffii	Pathological lesions in multiple organs and tissues, Bacteremia, pneumonia and meningitis,	Fish, Humans [51]
Kurthia gibsonii	Non-gonorrheal urethritis	Humans [52]
Sphingobacterium multivorum	Necrotizing fasciitis and septic shock, Bacteremia and acute meningitis	Humans [53,54]

References

1. Wang, W.; Cai, M.; Hu, J.; Zhang, Z.; Wang, X.; Chang, X.; Zhang, F.; Guo, C.; Wang, X. Mechanism of blood-brain barrier disruption by an *Escherichia coli* from lambs with severe diarrhea and meningoencephalitis. *Microb. Pathog.* **2020**, *147*, 104288, doi:10.1016/j.micpath.2020.104288.
2. Lima, P.C.; Dutra, I.D.S.; Araújo, F.A.; Lustosa, R.; Zeppelini, C.G.; Franke, C.R. First record of mass wild waterfowl mortality due to *Clostridium botulinum* in Brazilian semiarid. *Anais da Academia Brasileira de Ciências* **2020**, *92*, 20180370, doi:10.1590/0001-3765202020180370.
3. Xin, W.; Huang, Y.; Ji, B.; Li, P.; Wu, Y.; Liu, J.; Wang, X.; Yang, H.; Kang, L.; Gao, S.; An, X.P.; Xu, X.F.; Tong, Y.G.; Wang, J.L. Identification and characterization of *Clostridium botulinum* strains associated with an infant botulism case in China. *Anaerobe* **2019**, *55*, 1–7, doi:10.1016/j.anaerobe.2018.06.015.
4. Nocera, F.P.; Papulino, C.; Del Prete, C.; Palumbo, V.; Pasolini, M.P.; De Martino, L. Endometritis associated with *Enterococcus casseliflavus* in a mare: A case report. *Asian Pac. J. Trop. Biomed.* **2017**, *7*, 760–762, doi:10.1016/j.apjtb.2017.07.016.
5. Moreno, B.; Bolea, R.; Martín-Burriel, I.; Sanz-Rubio, D.; Romero, A.; Vazquez, F.J.; Badiola, J.J. Valvular endocarditis due to *Enterococcus casseliflavus* in a 4-month-old female foal. *J. Equine Veter- Sci.* **2014**, *34*, 1352–1356, doi:10.1016/j.jevs.2014.09.005.
6. Stubljar, D.; Skvarc, M. *Enterococcus cecorum* infection in two critically ill children and in two adult septic patients. *Slov. Vet. Res.* **2015**, *52*, 39–44.
7. Costa, M.O.; Lage, B. *Streptococcus equi* subspecies *zooepidemicus* and sudden deaths in Swine, Canada. *Emerg. Infect. Dis.* **2020**, *26*, 2522–2524, doi:10.3201/eid2610.191485.
8. Cárdenas, A.M.; Gomila, M.; Lalucat, J.; Edelstein, P.H. Abdominal abscess caused by *Mycobacterium llatzerense*. *J. Clin. Microbiol.* **2014**, *52*, 1287–1289, doi:10.1128/jcm.03525-13.
9. Teixeira, L.; Avery, R.K.; Iseman, M.; Arrossi, A.V.; Harrington, S.; Stephens, K.; Winans, C.G. *Mycobacterium llatzerense* lung infection in a liver transplant recipient: Case report and review of the literature. *Arab. Archaeol. Epigr.* **2013**, *13*, 2198–2200, doi:10.1111/ajt.12318.
10. Ramanan, P.; Deziel, P.; Razonable, R.R. *Rhodococcus globerulus* bacteremia in an allogeneic hematopoietic stem cell transplant recipient: report of the first transplant case and review of the literature. *Transpl. Infect. Dis.* **2014**, *16*, 484–489, doi:10.1111/tid.12220.
11. Töpfner, N.; Shetty, S.; Kunze, M.; Orlowska-Volk, M.; Krüger, M.; Berner, R.; Hentschel, R. Fulminant neonatal sepsis due to *Streptococcus alactolyticus* - A case report and review. *APMIS* **2014**, *122*, 654–656, doi:10.1111/apm.12219.
12. Angelakis, E.; Roux, V.; Raoult, D.; Drancourt, M. Human case of *Atopobium rimae* bacteremia. *Emerg. Infect. Dis.* **2009**, *15*, 354–355, doi:10.3201/eid1502.071399.
13. Mendes, R.E.; Denys, G.A.; Fritsche, T.R.; Jones, R.N. Case report of *Aurantimonas altamirensis* bloodstream infection. *J. Clin. Microbiol.* **2008**, *47*, 514–515, doi:10.1128/jcm.02171-08.

14. Principi, N.; Gnocchi, M.; Gagliardi, M.; Argentiero, A.; Neglia, C.; Esposito, S. Prevention of *Clostridium difficile* infection and associated diarrhea: an unsolved problem. *Microorg.* **2020**, *8*, 1640, doi:10.3390/microorganisms8111640.
15. Liu, Z.H.; Sossah, F.L.; Li, Y.; Fu, Y.P. First report of *Ewingella americana* causing bacterial brown rot disease on cultivated needle mushroom (*Flammulina velutipes*) in China. *Plant Dis.* **2018**, *102*, 2633, doi:10.1094/pdis-02-18-0351-pdn.
16. Ryoo, N.; Ha, J.S.; Jeon, D.S.; Kim, J.R.; Kim, H.C. A case of pneumonia caused by *Ewingella americanain* a patient with chronic renal failure. *J. Korean Med Sci.* **2005**, *20*, 143–145, doi:10.3346/jkms.2005.20.1.143.
17. Wenzler, E.; Kamboj, K.; Balada-Llasat, J.M. Severe sepsis secondary to persistent *Lysinibacillus sphaericus*, *Lysinibacillus fusiformis* and *Paenibacillus amylolyticus* bacteremia. *Int. J. Infect. Dis.* **2015**, *35*, 93–95, doi:10.1016/j.ijid.2015.04.016.
18. Bouchek-Mechiche, K.; Gardan, L.; Normand, P.; Jouan, B. DNA relatedness among strains of *Streptomyces* pathogenic to potato in France: Description of three new species, *S. europaeiscabiei* sp. nov. and *S. stelliscabiei* sp. nov. associated with common scab, and *S. reticuliscabiei* sp. nov. associated with netted scab. *Int. J. Syst. Evol. Microbiol.* **2000**, *50*, 91–99, doi:10.1099/00207713-50-1-91.
19. Javed, S.; Gul, F.; Javed, K.; Bokhari, H. *Helicobacter pullorum*: An emerging zoonotic pathogen. *Front. Microbiol.* **2017**, *8*, 604, doi:10.3389/fmicb.2017.00604.
20. Shah, R.; Klumpp, L.; Nerella, N.; Liu-Young, G.; Jordan, J. Infective endocarditis caused by *Rhodococcus equi* in an immunocompetent patient. *Cureus* **2020**, *12*, e7829, doi:10.7759/cureus.7829.
21. Suzuki, Y.; Takahashi, K.; Takase, F.; Sawada, N.; Nakao, S.; Toda, A.; Sasaki, Y.; Kakuda, T.; Takai, S. Serological epidemiological surveillance for vapN-harboring *Rhodococcus equi* infection in goats. *Comp. Immunol. Microbiol. Infect. Dis.* **2020**, *73*, 101540, doi:10.1016/j.cimid.2020.101540.
22. Hale, A.J.; Kirby, J.E.; Albrecht, M. Fatal spontaneous *Clostridium bifermentans* necrotizing endometritis: A case report and literature review of the pathogen. *Open Forum Infect. Dis.* **2016**, *3*, ofw095, doi:10.1093/ofid/ofw095.
23. Spigaglia, P.; Barbanti, F.; Marocchi, F.; Mastroleo, M.; Baretta, M.; Ferrante, P.; Caboni, E.; Lucioli, S.; Scortichini, M. *Clostridium bifermentans* and *C. subterminale* are associated with kiwifruit vine decline, known as moria, in Italy. *Plant Pathol.* **2020**, *69*, 765–774, doi:10.1111/ppa.13161.
24. Stes, E.; Francis, I.; Pertry, I.; Dolzblasz, A.; Depuydt, S.; Vereecke, D. The leafy gall syndrome induced by *Rhodococcus fascians*. *FEMS Microbiol. Lett.* **2013**, *342*, 187–195, doi:10.1111/1574-6968.12119.
25. Uysal, E.B.; Çelik, C.; Tuzcu, N.; Can, F.; Dogan, M.; Ertürk, R.; Bakici, M.Z. A case of chronic suppurative otitis media caused by *Kerstersia gyiorum*. *APMIS* **2015**, *123*, 986–989, doi:10.1111/apm.12434.
26. Bostwick, A.D.; Zhang, C.; Manninen, K.; Touchberry, J.; Greene, S.R.; Holland, T.L. Bacteremia caused by *Kerstersia gyiorum*. *J. Clin. Microbiol.* **2015**, *53*, 1965–1967, doi:10.1128/jcm.03625-14.
27. Hammer, A.; Wolff, D.; Geißdörfer, W.; Schrey, M.; Ziegler, R.; Steiner, H.H.; Bogdan, C. A spinal epidural abscess due to *Streptobacillus moniliformis* infection following a rat bite: case report. *J. Neurosurgery: Spine* **2017**, *27*, 92–96, doi:10.3171/2016.12.spine161042.
28. Hayakawa, Y.; Suzuki, J.; Suzuki, M.; Sugiura, W.; Ohkusu, K. A case study of rat bite fever caused by *Streptobacillus moniliformis*. *Jpn. J. Infect. Dis.* **2017**, *70*, 323–325, doi:10.7883/yoken.JJID.2016.270.
29. Capewell, P.; Rupp, A.; Fuentes, M.; McDonald, M.; Weir, W. Fatal *Clostridium sordellii*-mediated hemorrhagic and necrotizing gastroenteropathy in a dog: Case report. *BMC Veter- Res.* **2020**, *16*, 1–5, doi:10.1186/s12917-020-02362-y.
30. Bravo-Ojeda, J.; Gomez-Quintero, C.; Pescador-Vargas, L.; Suarez-Tirado, J. Bacteriemia por *Clostridium sordellii* en paciente con neoplasia gastrointestinal. Reporte de caso y revisión de literatura. *Infect.* **2020**, *24*, 133–137, doi:10.22354/in.v24i2.846.
31. Hendriksen, S.W.M.; Leengoed, L.A.M.G.V.; Roest, H.I.J.; Van Nes, A. Neonatal diarrhoea in pigs: alpha- and beta2-toxin produced by *Clostridium perfringens*. *Tijdschr. voor Diergeneeskd.* 2006, *131*, 910–913.
32. Noël, A.; Verroken, A.; Belkhir, L.; Rodriguez-Villalobos, H. Fatal thoracic empyema involving *Campylobacter rectus*: A case report. *Anaerobe* **2018**, *49*, 95–98, doi:10.1016/j.anaerobe.2017.12.014.
33. Kakuta, R.; Hidaka, H.; Yano, H.; Okamoto, M.; Ozawa, D.; Endo, S.; Kaku, M.; Katori, Y. First report of severe acute otitis media caused by *Campylobacter rectus* and review of the literature. *J. Infect. Chemother.* 2016, *22*, 800–803.
34. Vendrell, D.; Balcázar, J.L.; Ruiz-Zarzuela, I.; De Blas, I.; Gironés, O.; Múzquiz, J.L. *Lactococcus garvieae* in fish: A review. *Comp. Immunol. Microbiol. Infect. Dis.* **2006**, *29*, 177–198, doi:10.1016/j.cimid.2006.06.003.
35. Kim, H.J.; Yunkyoung, L.; Kyunghwan, O.; Sang-Ho, C.; Heungsup S.; Jin, W.H. Septic shock due to unusual pathogens, *Comamonas testosteroni* and *Acinetobacter guillouiae* in an immune competent patient. Korean *J. Crit. Care Med.* **2015**, *30*, 180–183.
36. Sridhar, S.; Wang, A.Y.M.; Chan, J.F.W.; Yip, C.C.Y.; Lau, S.K.P.; Woo, P.C.Y.; Yuen, K.Y. First report of human infection by *Agromyces mediolanus*, a gram-positive organism found in soil. *J. Clin. Microbiol.* **2015**, *53*, 3377–3379, doi:10.1128/jcm.01508-15.
37. Jeon, Y.; Kim, T.S.; Bin Kim, H.; Park, K.U.; Song, J.; Kim, E.C. First Korean case of *Robinsoniella peoriensis* bacteremia in a patient with aspiration pneumonia. *Ann. Lab. Med.* **2012**, *32*, 370–374, doi:10.3343/alm.2012.32.5.370.
38. Ruiz-Zarzuela, I.; De Bias, I.; Girones, O.; Ghittino, C.; Múazquiz, J.L. Isolation of *Vagococcus salmoninarum* in rainbow trout, *Oncorhynchus mykiss* (Walbaum), broodstocks: characterization of the pathogen. *Veter- Res. Commun.* **2005**, *29*, 553–562, doi:10.1007/s11259-005-2493-8.

39. Gubler, J.G.; Wuest, J.; Hany, A. Pleuropulmonary infection due to *Clostridium subterminale*. *J. Infect.* **1989**, *19*, 277–280, doi:10.1016/s0163-4453(89)90849-9.
40. Hu, Y.; Zheng, D.; Takizawa, K.; Mikami, Y.; Dai, L.; Yazawa, K.; Fukushima, K.; Lu, C.; Xi, L. Systemic nocardiosis caused by *Nocardia concavain* in China. *Med Mycol.* **2011**, *49*, 1–5, doi:10.3109/13693786.2011.555849.
41. Hirayama, T.; Takazono, T.; Horai, Y.; Tashiro, M.; Saijo, T.; Kosai, K.; Morinaga, Y.; Kurihara, S.; Nakamura, S.; Imamura, Y.; Miyazaki, T.; Tsukamoto, M.; Izumikawa, K.; Yanagihara, K.; Kawakami, A.; Kohno, S. Pulmonary nocardiosis caused by *Nocardia concava* with a literature review. *Intern. Med.* **2016**, *55*, 1213–1217, doi:10.2169/internalmedicine.55.6228.
42. Jaime-Villalonga, A.; Saul, Z.; Miljkovic, G. *Mycobacterium arupense* finger osteomyelitis: case report. *Int. J. Infect. Dis.* **2020**, *92*, 226–227, doi:10.1016/j.ijid.2020.01.027.
43. Lopez, F.K.; Miley, M.; Taiwo, B. *Mycobacterium arupense* as an emerging cause of tenosynovitis. *Emerg. Infect. Dis.* **2016**, *22*, 559–561, doi:10.3201/eid2203.151479.
44. Malik, W.; McLeod, G. Persistent *Elizabethkingia meningoseptica* bacteremia in a patient with multiple myeloma. *IDCases* **2019**, *18*, e00617, doi:10.1016/j.idcr.2019.e00617.
45. Mathur, P.; Varghese, P.; Misra, M.C.; Tak, V. *Elizabethkingia meningoseptica*: An emerging pathogen causing meningitis in a hospitalized adult trauma patient. *Indian J. Med Microbiol.* **2013**, *31*, 293–295, doi:10.4103/0255-0857.115653.
46. Mirghasempour, S.A.; Shiwen, H.; Xie, G.L. First report of *Burkholderia gladioli* causing rice panicle blight and grain discoloration in China. *Plant Dis.* **2018**, *102*, 2635, doi:10.1094/pdis-05-18-0758-pdn.
47. You, Y.; Lü, F.B.; Zhong, R.H.; Chen, H.M.; Li, H.P.; Liu, J.M.; Zhang, J.H. First report of bacterial brown spot in *Phalaenopsis* spp. caused by *Burkholderia gladioli* in China. *Plant Dis.* **2016**, *100*, 1232, doi:10.1094/pdis-09-15-0963-pdn.
48. Ogasawara, M.; Matsuhisa, T.; Kondo, T.; Oshima, R.; Sugiura, F.; Niwa, T.; Ando, Y.; Sato, M.; Sato, J.; Kohri, S. Pyogenic spondylitis with acute course caused by *Corynebacterium simulans*. *J. Infect. Chemother.* **2020**, *26*, 294–297, doi:10.1016/j.jiac.2019.10.012.
49. Garcia-Gonzalez, E.; Genersch, E. Honey bee larval peritrophic matrix degradation during infection with *Paenibacillus* larvae, the aetiological agent of American foulbrood of honey bees, is a key step in pathogenesis. *Environ. Microbiol.* **2013**, *15*, 2894–2901, doi:10.1111/1462-2920.12167.
50. Jansåker, F.; Bollestad, M.; Vik, I.; Lindbaek, M.; Bjerrum, L.; Frimodt-Møller, N.; Knudsen, J.D. Pivmecillinam for uncomplicated lower urinary tract infections caused by *Staphylococcus saprophyticus*—cumulative observational data from four recent clinical studies. *Antibiot.* **2019**, *8*, 57, doi:10.3390/antibiotics8020057.
51. Cao, S.; Geng, Y.; Yu, Z.; Deng, L.; Gan, W.; Wang, K.; Ou, Y.; Chen, D.; Huang, X.; Zuo, Z.; et al. *Acinetobacter lwoffii*, an emerging pathogen for fish in *Schizothorax* genus in China. *Transbound. Emerg. Dis.* **2018**, *65*, 1816–1822, doi:10.1111/tbed.12957.
52. Ongrádi, J.; Stercz, B.; Kövesdi, V.; Nagy, K.; Chatlynne, L. Isolation of *Kurthia gibsoniifrom* non-gonorrheal urethritis: implications for the pathomechanism upon surveying the literature. *Acta Microbiol. et Immunol. Hung.* **2014**, *61*, 79–87, doi:10.1556/amicr.61.2014.1.8.
53. Grimaldi, D.; Doloy, A.; Fichet, J.; Bourgeois, E.; Zuber, B.; Wajsfisz, A.; Mira, J.P.; Poyart, C.; Pène, F. Necrotizing fasciitis and septic shock related to the uncommon gram-negative pathogen *Sphingobacterium multivorum*. *J. Clin. Microbiol.* **2011**, *50*, 202–203, doi:10.1128/jcm.05151-11.
54. Abro, A.H.; Shahmirzadi, M.R.R.; Jasim, L.M.; Badreddine, S.; Al Deesi, Z. *Sphingobacterium multivorum* bacteremia and acute meningitis in an immunocompetent adult patient: A case report. *Iran. Red Crescent Med J.* **2016**, *18*, e38750, doi:10.5812/ircmj.38750.
